# Adaptive Visual Sensing Deviation Detection and Real-Time Tracking Control of Swing-Arc Narrow-Gap Weld Based on Variation Coefficient Recognition

**DOI:** 10.3390/s26175336

**Published:** 2026-08-23

**Authors:** Jie Wang, Na Su, Jiayou Wang

**Affiliations:** 1 The School of Communications and Information Engineering, Nanjing University of Posts and Telecommunications, Nanjing 210003, China; 2 The School of Materials Science and Engineering, Jiangsu University of Science and Technology, Zhenjiang 212003, China; jywang@just.edu.cn

**Keywords:** narrow-gap welding, visual sensing, variation coefficient, weld deviation detection, weld tracking

## Abstract

**Highlights:**

**What is the main finding?**

- An in situ bandpass data filter based on variation coefficient minimization adaptively recognizes the real position of a groove center.

**What are the implications of the main findings?**

- A data window of self-adaptive height lowers the variation degree of the raw groove-centerline data distribution to further improve the detection accuracy.
- A real-time swing-arc narrow-gap weld tracking system achieves higher accuracy within ±0.2 mm based on infrared passive vision sensing.

**Abstract:**

Weld tracking aims to provide real-time compensation for weld deviations caused by groove assembly inaccuracies and thermal shrinkage during Gas Metal Arc Welding (GMAW). To achieve precise tracking control in swing-arc narrow-gap GMAW based on passive visual sensing, a Self-Adaptive Coefficient-of-Variation Recognition (SCVR) algorithm is proposed for adaptively detecting weld deviation by filtering out welding interference. SCVR adaptively constructs a data window by discriminating the original variation coefficient to acquire the raw data distribution of the groove centerline. It then designs an in situ bandpass data filter to locally search the data segment with the minimal coefficient of variation for adaptive bandwidth determination. By applying the filter to the raw data, the disturbed data are removed, in situ retaining the data with the globally minimized variation coefficient. Finally, SCVR recognizes the real groove center from the filtered data, accurately detecting a weld deviation by comparing this center to the torch position. Additionally, an SCVR-based real-time tracking control system incorporating a PLC-based actuator with a PI controller for optimal stability is developed to correct the torch position in real time, achieving a high tracking precision of −0.161~+0.126 mm. Experimental results demonstrate the robust adaptability and effectiveness of the SCVR-based weld detection and tracking control system.

## 1. Introduction

As an efficient process for thick-walled steel structure manufacturing, swing-arc narrow-gap Gas Metal Arc Welding (GMAW) has gained particular attention, owing to the outstanding directivity and motion-parameter controllability of the arc and the suitability of the process for all-position welding [1,2,3]. However, during narrow-gap welding, a number of factors, including the welding groove assembly inaccuracy and thermal shrinkage, usually cause the welding torch to shift from the groove center, resulting in weld deviation [4,5]. This deviation leads to insufficient and nonuniform penetration into the groove sidewalls, ultimately lowering welding quality [6]. To elevate the quality and the automatic and intelligent level of the welding process, therefore, weld tracking to compensate in real time for weld deviation is crucial.

The sensing and detection of weld deviation are the keys to weld tracking. The current sensing methods mainly comprise visual sensing [7,8,9], arc sensing [10,11], and tactual sensing [12]. Among these, visual sensing [13,14,15], including the active and passive types, stands out due to its advantages in noncontact detection, the ability to capture detailed information, high detection accuracy, and strong anti-interference capabilities. Notably, compared to active visual sensing, passive visual sensing enables synchronous detection of weld deviation with the arc position without the need for an additional light source, leading to higher detection accuracy and better robustness. However, there still exist challenges in visual sensing, especially in narrow-gap arc welding [16], such as the disturbances of a strong arc light, welding spatter, and welding fumes. The disturbances degrade the detection precision of the weld deviation, in addition to influencing the monitoring of welding quality [17,18].

To suppress disturbances, two aspects of measures are commonly taken from the sensing technique and detection algorithm. Typically, several sensing techniques are applied to decrease arc-light interference, such as passive visual sensing [15], the narrowband and neutral filtering system [19,20], and image-capturing devices externally triggered by the electrical and/or positional signals of the welding arc [16,21]. In addition, image processing and deviation detection algorithms have been developed to further reduce welding disturbances.

On the one hand, efforts have been carried out to increase the robustness of edge extraction. During passive-visual GMAW image processing, Xu et al. [22] improved a Canny algorithm to accurately acquire the edges of the seam and pool from an image, realizing seam tracking. Ye et al. [23] proposed a robust image processing method to extract the centers of the welding pool and butt seam from an ROI window image, gaining a weld offset from the image containing much random noise. To further increase the accuracy of edge extraction, Banafian et al. [24] improved the Laplacian and Median filtering-based image processing algorithms to precisely extract the edges of curved seams and the robotic welding pool for butt-joint seam tracking. However, all the above methods determine the pool center from the horizontal terminal points of the pool contour, which influences the detection accuracy of weld deviation due to contour irregularity. Accordingly, the three methods provide accuracy in the range of ±0.3~±1.03 mm.

On the other hand, to improve the accuracy of deviation detection, several detection algorithms have been designed to further reduce welding interference in extracting edges. Su et al. [16] proposed an outlier data filtering (ODF) detection approach to reduce the effect of interference during swing-arc narrow-gap GMAW. However, ODF reduces adaptability to varying-level interference while increasing detection time, due to using a fixed large ROI window size of 80 × 80 pixels. Additionally, ODF directly extracts the two groove edges, rather than the groove centerline, and thus is more sensitive to interference at the two edges. Moreover, Xia et al. [25] used a local-feature moving-window algorithm to measure the groove center and realized weld tracking with a lower accuracy of ±0.47 mm. Zhu et al. [26] proposed a local pattern recognition (LPR) algorithm to locally search the straightest segment of a groove edge to detect the groove center. Both of the latter two methods are readily trapped in local optima because of their use of local pattern recognition. Thus, the above three methods have lower adaptivity to the welding environment, in addition to increasing the detection time.

Additionally, in non-consumable arc-weld tracking based on passive visual sensing, Zhang et al. [27] improved a Hough-based algorithm with a position-adaptive ROI window in GTAW, achieving a high seam-detection accuracy within ±0.04 mm. However, the Hough algorithm suffers from certain limitations, including prolonged computational time and limited adaptability to complex welding environments. Lin et al. [28] used object tracking to locate the keyhole entrance, enabling seam tracking in super-narrow-gap GTAW. Hong et al. [29] applied spatial–temporal deep learning on molten pool images for real-time tracking in micro-plasma arc butt-joint welding. Nevertheless, the above detection algorithms are not capable of resisting strong welding disturbances because the stability of the non-consumable process is much better than that of the GMAW process.

According to the above analyses, the existing image processing and weld deviation detection algorithms lack optimal adaptability in GMAW processes. Particularly in narrow-gap arc welding with a narrow and deep groove, welding spatters of various sizes more easily attach to the groove sidewalls, while more welding fumes and arc light accumulate inside the groove. These random welding disturbances with variant levels pose challenges for the detection of welding features. To address the limitations, an adaptive visual sensing weld deviation detection and real-time tracking control system is proposed for promising swing-arc narrow-gap welding. Specifically, a Self-adaptive Coefficient-of-Variation Recognition (SCVR) algorithm is proposed to adaptively determine the groove center by globally minimizing variation coefficients in filtering out welding interference. The SCVR-based approach aims to precisely detect weld deviation from the groove-torch positional difference while reducing detection time, enabling real-time high-accuracy weld tracking using infrared vision.

The main contributions of the method include the following: (1) An in situ bandpass data-filtering detection method based on variation coefficient minimization is proposed in SCVR to adaptively recognize the real position of the groove center by filtering out the disturbed data, enabling precise measurement of weld deviation. (2) A data window of adaptive height adjustment is established to lower the proportion of the disturbed data within the raw groove centerline by discriminating the original variation coefficient, further improving the detection robustness of SCVR. (3) An SCVR-based real-time control system, incorporating a PLC-based actuator with a PI controller of optimal stability, is developed to achieve higher weld-tracking accuracy in narrow-gap welding.

This work helps to elevate the quality and intelligence level of narrow-gap welding, further advancing industrial applications of the welding process. Additionally, it provides an algorithmic reference for broader applications in pattern recognition.

## 2. Experimental System and Image Processing

### 2.1. System Implementation

**Figure 1:**
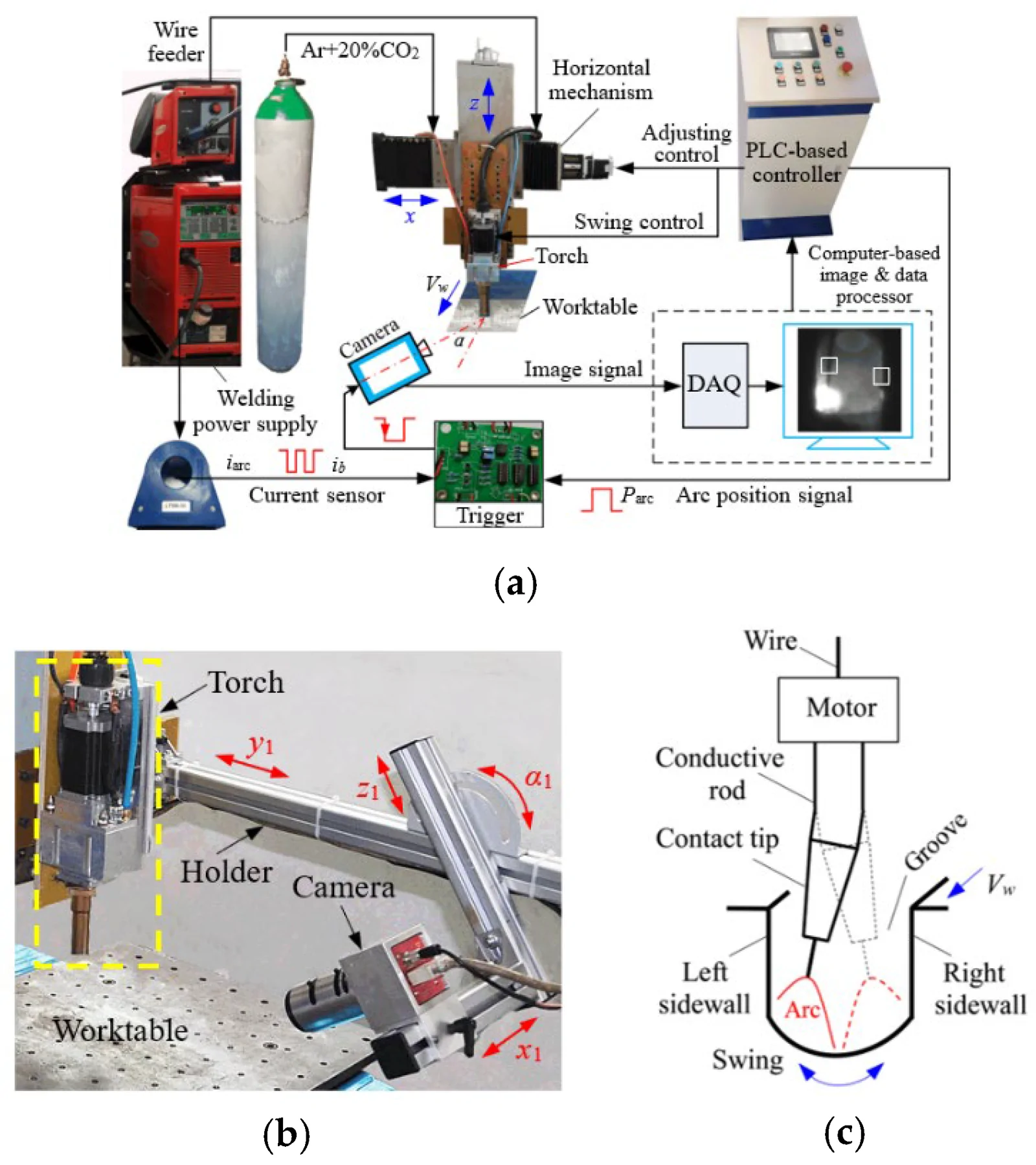
Schematic diagram of infrared passive vision-based weld deviation detection and real-time tracking control system for swing-arc narrow-gap welding. (**a**) System configuration. (**b**) Photograph of camera mounting setup [30]. (**c**) Illustration of arc swing [1].

Figure 1 shows a schematic diagram of the experimental system, which includes four parts: a swing-arc narrow-gap welding setup, a manipulator, a visual sensing detection unit, and a PLC-based controller. This welding setup contains a welding power supply, a wire feeder, a swing-arc welding torch, and a shielding gas supplier, as shown in Figure 1a. The positive and negative terminals of the welding power supply are electrically connected to the torch and the testpiece. The manipulator is designed to laterally (*V_w_*-directionally) move a testpiece on the worktable, vertically (*z*-axially) regulate the torch standoff height, and locate the torch horizontal (*x*-axial) position. Moreover, the visual sensing detection unit comprises a Photonfocus MV1-D1312I infrared 8-bit CMOS camera, an external trigger for image capturing, and the image and data processor in a computer. It functions to process welding images and output an adjustment signal for the torch position in the processor, as well as capturing images. To demonstrate the actual position of the camera, Figure 1b shows a photograph of the camera mounting setup. A holder with three straight rails and one rotatable joint fixes the camera onto the torch. Accordingly, the camera positions can be regulated along the straight-line directions of *x*_1_, *y*_1_, and *z*_1_, while the inclination angle of the camera is adjusted along the angular direction of *α*_1_ via the joint.

In welding, the PLC-based controller governs the arc to periodically swing within the groove, as shown in Figure 1c. The arc swing parameters include the swing angle, the frequency, and the staying (dwell) time at the groove sidewalls. Simultaneously, the controller drives the manipulator to move the testpiece at a welding speed of *V_w_* relative to the torch. As the arc swings to stay at any groove sidewall, the PLC-based controller outputs an arc position signal, *P*_arc_. Simultaneously, the current sensor detects the base-value signal, *i_b_*, of the pulsed arc current, *i*_arc_. The trigger then combines *P*_arc_ with *i_b_* to externally activate the camera to capture a welding image at any extreme position of the swing. Subsequently, the processor analyzes the images transmitted via a data acquisition (DAQ) card to extract weld deviation and outputs an adjustment value for the torch position to the PLC-based controller, enabling real-time weld tracking.

### 2.2. Image Capturing and Processing

#### 2.2.1. Image Capturing

**Figure 2:**
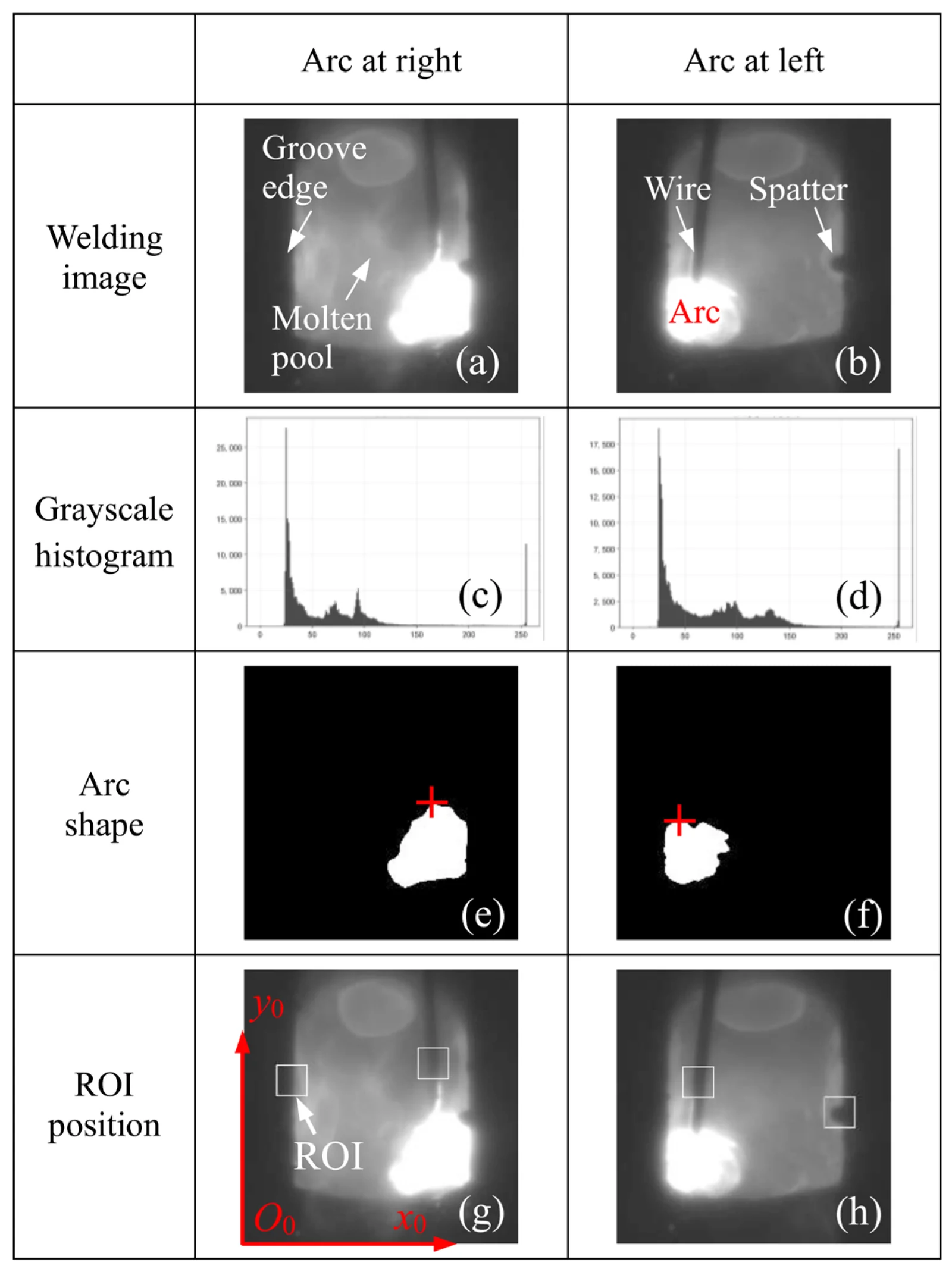
Examples of self-adaptively positioning the ROI windows. (**a**,**b**) Two adjacent welding images at the right and left positions of the arc. (**c**,**d**) Grayscale histogram of welding image. (**e**,**f**) Arc shapes with the highest points indicated by red-crossing signs. (**g**,**h**) Adaptively located ROI windows of the groove edge and the wire.

To acquire welding images, the camera focuses downwards on the molten pool in front of the torch at an inclination angle, α (see Figure 1a), of ~30°. To reduce the light intensities of the welding arc and molten pool while protecting the camera lens, a composite optical filter is installed in front of the camera lens. This filter successively consists of a narrowband filter with a wavelength of 970 ± 20 nm, a neutral density filter with a transmittance ratio of 30%, and a protective glass. By integrating this optical filter with the aforementioned external trigger, the passive infrared sensing method can significantly reduce the disturbances from welding fumes and the arc light. As a result, a high-quality infrared welding image with a size of 544 × 544 pixels is obtained, as exemplified in Figure 2a,b, as well as Figure 1a, in which the arc, molten pool, and electrode wire are clearly visible. The related parameters of the welding experiments and camera shooting are listed in Table 1 and Table 2, and the torch was aligned with the groove center.

#### 2.2.2. Image Processing

The extraction of features from welding images necessitates a preliminary preprocessing step. To raise the processing efficiency in extracting the groove edges and the welding wire contour, a region-of-interest (ROI) window is used to locally intercept the welding images. Adaptive positioning of the ROI window is essential for accurate extraction, as welding is a dynamic process where weld deviation, groove-width change, and random arc fluctuation occur.

**(a) ROI window localization**. The longitudinal and horizontal positions of the ROI window are located respectively by the arc highest point and the groove-edge position. To search for this highest point, a global binarization threshold is first established. This threshold is selected as 250 by performing a grayscale histogram analysis on a series of sample welding images, such as Figure 2c,d, ensuring a stable arc morphology. The welding image is then processed globally through median filtering, followed by binarization using the threshold. Subsequently, the coordinates of the arc highest point are identified via an adaptive and row-by-row scan of the binary image.

Consequently, the ROI windows for the groove edge and the wire can be longitudinally positioned based on the highest point, where the longitudinal direction corresponds to the *y*_0_-axial direction of the global welding image in Figure 2. The horizontal (i.e., *x*_0_-axial in Figure 2) position of the current groove-edge ROI window is then adaptively located according to its same-side edge position in the prior frame, thereby centering the groove edge within the current window. The self-adaptive positioning is finally realized for both ROI windows. Similarly, the current ROI window of the wire is positioned by pre-welding horizontal calibration and the vertical coordinate of the current arc highest point. Figure 2 shows two examples of self-adaptive ROI window positioning. Notably, the ROI window of the groove edge is always placed on the opposite side of the arc to reduce the interference of the arc light.

**(b) ROI image feature extraction**. To measure weld deviation from the groove center and torch position, the groove edges and wire contour are extracted by processing their respective ROI images [31]. Firstly, a raw groove edge is obtained by sequentially processing its ROI image with median filtering [32], contrast enhancement by piecewise linear stretch, Otsu thresholding, morphological closing operation [33] with a 3 × 3 rectangular structuring element, and a Canny operator [34]. To extract two contour lines of the wire, the ROI image of the wire is then processed in turn by local adaptive thresholding, a morphological opening operation with a 3 × 3 rectangular structuring element, and a Canny operator. As a result, the raw centerlines of the groove and the wire, which contain welding noise, are respectively calculated from the two extracted groove edges and two wire-contour centerlines.

**Figure 3:**
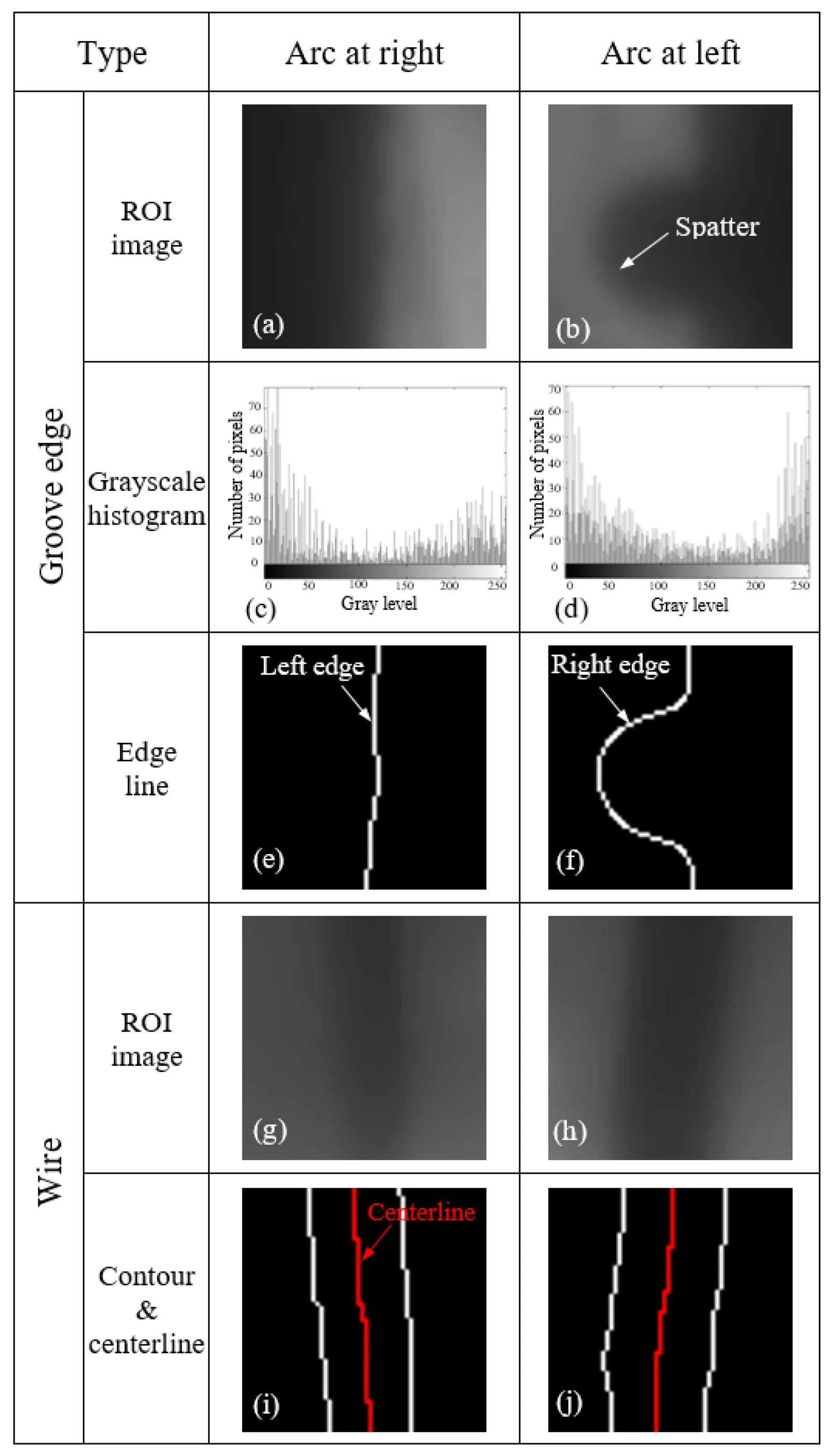
Extracted examples of the groove edge and wire contour. (**a**,**b**) ROI images of the left and right groove edges at the right and left positions of the arc. (**c**,**d**) Grayscale distribution of the stretched ROI images. (**e**,**f**) Left and right groove edges extracted. (**g**,**h**) ROI images of the wire at the right and left positions of the arc. (**i**,**j**) Red wire centerlines calculated by averaging the two white wire contours.

Figure 3 shows examples of the feature extraction of the groove edge and wire contour, where the size of each ROI window is 60 × 60 pixels. The four ROI images in Figure 3a,b,g,h were intercepted from the corresponding white ROI windows in Figure 2g,h. Figure 3c,d illustrate the grayscale distribution of the left and right groove-edge images after stretching the images. This stretching significantly extends the grayscale range from <150 to 255 levels.

Subsequently, the Otsu method is applied to perform thresholding on the image with a bimodal distribution. After the thresholding, the raw groove edges are extracted and indicated by the white lines in Figure 3e,f. On the right edge line of the groove in Figure 3f, a large actual welding spatter of ~1.2 mm diameter clearly exists, which increases the difficulty of accurately detecting the groove center.

In addition, two pairs of white lines in Figure 3i,j represent the extracted contour lines of the wire. Accordingly, the centerlines of the wire at the two positions are calculated by averaging the two contour lines, as indicated by the red lines in Figure 3i,j.

## 3. Weld Deviation Detection Approach

### 3.1. Principle of Weld Deviation Detection

By calculating the positional difference between the detection values for the groove center and the torch position from two consecutive frames of welding images, a weld deviation value can be obtained. Owing to the randomness of the welding disturbances, each detection value for the groove center and torch position is actually derived by the following filtering algorithms. Consequently, even if the sampled values for the groove center and the torch position fail to effectively exclude the disturbances exceeding the limitations of the sampling algorithms (e.g., SCVR and SATV), the filtering algorithms can subsequently filter out the escaping disturbances to gain the detection values.

Specifically, the sampling algorithm of the torch position (SATV) yields a sampled value of the torch position by directly averaging all the data points on the left and right centerlines of the wire. A detection value, ${\hat{x}}_{i}^{\left(e\right)}$, of the torch position is then estimated by applying the PCF (Position Consistency Filtering) algorithm [16]. Additionally, to gain a sampled value of the groove center, a sampling algorithm using the Self-adaptive Coefficient-of-Variation Recognition (SCVR) is developed below to filter out the disturbed data in the raw data distribution of the groove centerline. Subsequently, a detection value, ${\hat{x}}_{i}^{\left(g\right)}$, of the groove center is acquired after using the amplitude-limiting mean filtering (ALMF) algorithm [16], in which the most recent several sampled values are applied. Finally, the current weld deviation, Δ*x_i_*, for the *i*-th sampling is directly detected as

(1) $${\Delta x}_{i}\;=\;({\hat{x}}_{i}^{\left(g\right)}\;-\;{\hat{x}}_{i}^{\left(e\right)})$$

### 3.2. Algorithm of Groove-Center Detection

#### 3.2.1. Algorithm Principle

Through several techniques (e.g., compound optical filtering, external triggering, and arc-opposite imaging; see Section 2.2), the infrared camera apparently reduces disturbances from the arc light and welding fumes. Even so, such interference, probably together with welding spatter, still remains on the welding images to varying extents. Subsequent ROI image processing (Section 2.2.2) serves to extract groove edges with enhanced image quality but cannot fully eliminate these disturbances on the edge lines, resulting in non-straight groove-edge lines in Figure 3e,f. Accordingly, the groove centerline, derived from the two edge lines, becomes locally curved.

**Figure 4:**
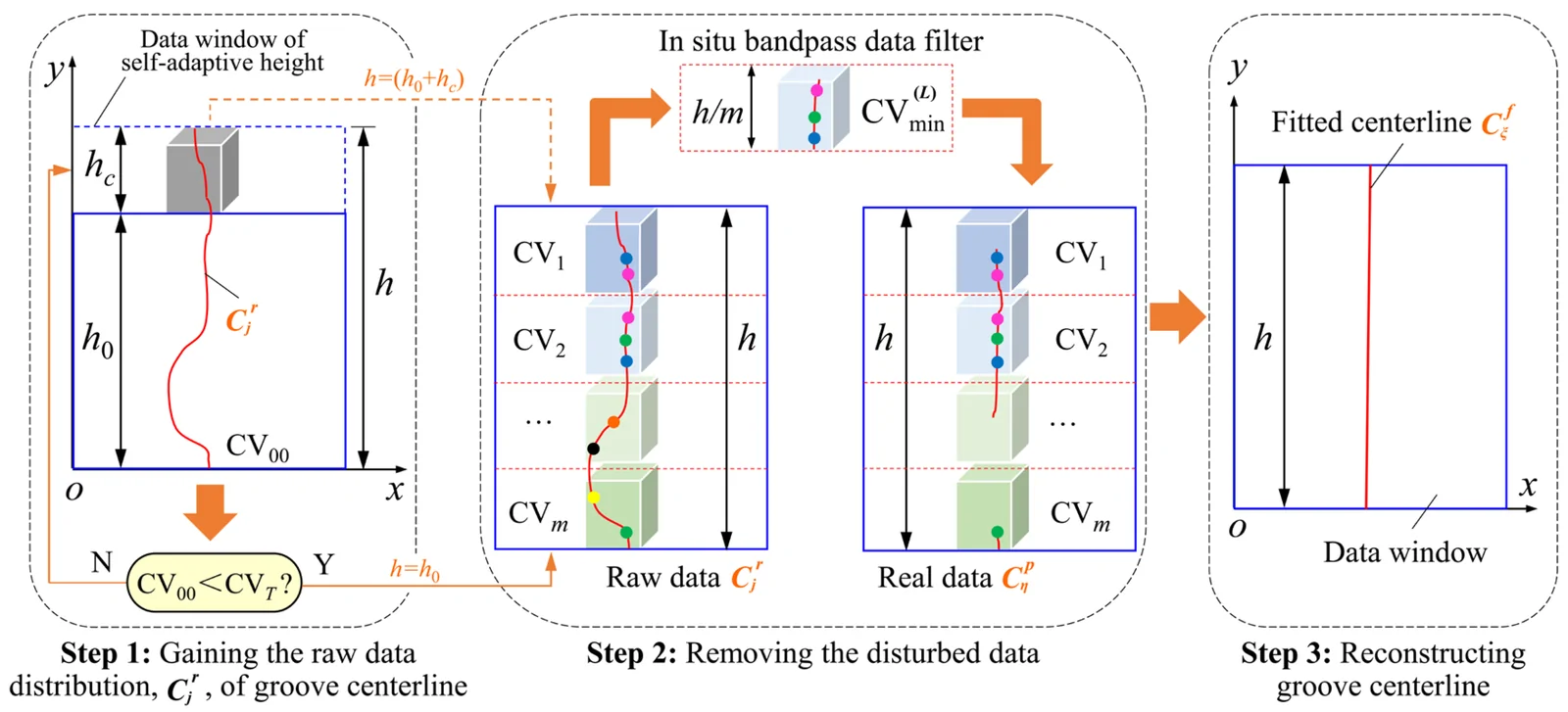
Principal diagram of SCVR algorithm.

To detect the accurate groove center, a Self-adaptive Coefficient-of-Variation Recognition (SCVR) algorithm is proposed. The principal diagram of the SCVR algorithm is presented in Figure 4. SCVR incorporates a mechanism of self-adaptively heightening the data window of the groove centerline and an in situ self-adaptive bandpass data filter that removes disturbance components. As the level of the welding disturbance varies, the coefficient of variation (CV) changes. Accordingly, the self-adaptively heightening and the self-adaptive bandpass data filter perform, self-adaptively removing the influence of the disturbance at varying levels. SCVR comprises three steps: gaining the raw data distribution of the groove centerline, removing the disturbed data, and reconstructing the groove centerline.

***Step* 1: Gaining the raw data distribution**. SCVR constructs a data window with self-adaptive height to resist random welding interference at varying levels. As illustrated in Figure 4, a raw data distribution, $C_{j}^{r}$, of the groove centerline contains *h* data points in the data window with self-adaptive height. This distribution is derived by averaging the data points of the left and right groove-edge lines obtained in Section 2.2. Accordingly, a raw data distribution matrix, $C_{j}^{r}$ = $\left[g_{j}\right]$, forms, where ***r*** and ***j*** respectively denote the *raw data* and *data number* in this matrix, $g_{j}$ is the *j*-th element in the matrix, and *j* = 1, 2,…, *h*.

The height, *h* (in pixels), of the data window is determined via a self-adaptation mechanism to mitigate the impact of welding disturbances on the detection accuracy of the groove center. Accordingly, the coefficient of variation (CV_00_) of the *h*_0_ data points is calculated from the initial raw data distribution. If CV_00_ < CV*_T_*, the height, *h*, remains an initial height, *h*_0_ (in pixels); otherwise, *h* increases from *h*_0_ to (*h*_0_ + *h_c_*) via varying the ROI height to reduce the extent of interference, where *h_c_* is a set value. CV*_T_* is a threshold for the coefficient of variation. Here, *h* and CV_00_ are expressed as

(2) $$h=\{\begin{array}{cc} h_{0} & \left({\mathrm{CV}}_{00}\;<\;{\mathrm{CV}}_{T}\right)\\ h_{0}+h_{c} & \left({\mathrm{CV}}_{00}\;\geq \;{\mathrm{CV}}_{T}\right) \end{array}$$

(3) $${\mathrm{CV}}_{00}=\frac{1}{{\overset{-}{g}}_{0}}\cdot \sqrt{\frac{1}{h_{0}}\sum_{j=1}^{h_{0}}{\left(g_{j}\;-\;{\overset{-}{g}}_{0}\right)}^{2}\;}$$

where $g_{j}$ and *j* are respectively the *x*- and *y*-coordinates of the *j*-th raw data point on the groove centerline and ${\overset{-}{g}}_{0}$ denotes the mean value of the *h*_0_ data in the data window. The value of *h_c_* is determined as established by the following simulated spatter and actual welding experiments.

***Step* 2: Removing the disturbed data**. An in situ bandpass data filter is designed to *locally* identify the data segment with the minimal coefficient of variation (${\mathrm{CV}}_{\min}^{\left(L\right)}$) from the raw data distribution, which adaptively sets the filter bandwidth. The filter then retains the data within this bandwidth in situ while removing out-of-range disturbances. The remaining data, called the real data, yield a *global* minimal coefficient of variation, ${\mathrm{CV}}_{\min}^{\left(G\right)}$. The removed data are treated as disturbed data, which result from random interference, primarily including welding spatter, the arc light, or welding fumes.

Specifically, the raw data distribution of the groove centerline (from *Step* 1) is further divided into *m* segments at the height, *h*, of the data window. For every raw data segment, each variation coefficient, CV*_ε_* (*ε* = 1, 2,…, *m*), is calculated individually. The minimum value, ${\mathrm{CV}}_{\min}^{\left(L\right)}$, among these *m* coefficients is then recognized. The value range of the data segment with ${\mathrm{CV}}_{\min}^{\left(L\right)}$ defines the lower and upper cutoff values for the bandpass data filter, thereby determining its bandwidth, *D*_width_. This filter removes the disturbed data outside, *D*_width_.

The preserved data form the in situ real-data distribution matrix $C_{\eta }^{p}$ = $\left[g_{j}\right]$ (*j*∈[1, *h*]), where ***p*** and ***η*** (***η*** ≤ *h*) respectively denote the *preserved data* and *data number* in $C_{\eta }^{p}$. Additionally, the subscript *j* in the data element $g_{j}$ represents the original ordinal number of the element in the raw data distribution matrix, $C_{j}^{r}$. This matrix, $C_{\eta }^{p}$, is obtained after globally filtering out the disturbed data, thus realizing a globally optimal selection of the real data. Accordingly, CV*_ε_*, ${\mathrm{CV}}_{\min}^{\left(L\right)}$, and *D*_width_ are defined as

(4) $${\mathrm{CV}}_{\varepsilon }=\frac{1}{{\overset{-}{g}}_{\varepsilon }}\cdot \sqrt{\frac{1}{h_{s}}\sum_{k=1}^{h_{s}}{\left(g_{k}\;-\;{\overset{-}{g}}_{\varepsilon }\right)}^{2}}$$

(5) $${\mathrm{CV}}_{\min}^{\left(L\right)}=\min\;\{{\mathrm{CV}}_{\varepsilon }\;\};\;\varepsilon \;=1,\;2,\;\ldots ,m$$

(6) $$D_{\mathrm{width}}\;=\;[{\min\{g_{k}\}|}_{{\mathrm{CV}}_{\varepsilon }={\mathrm{CV}}_{\min}^{\left(L\right)}},\;{\max\{g_{k}\}|}_{{\mathrm{CV}}_{\varepsilon }={\mathrm{CV}}_{\min}^{\left(L\right)}}]$$

where $g_{k}$ is the *x*-coordinate of the *k*-th raw data point in the *ε*-th segment, ${\overset{-}{g}}_{\varepsilon }$ denotes the mean value of the *h_s_* data in the *ε*-th segment, and *h_s_* represents the height of each segment. Accordingly, *h_s_* = *h/m*; *k* = 1, 2,…, *h_s_*; and *ε* = 1, 2,…, *m*.

***Step* 3: Reconstructing the groove centerline**. SCVR reconstructs the groove centerline by linearly fitting the real data retained from *Step* 2. The fitted-data distribution matrix $C_{\xi }^{f}$ = $\left[g_{\xi }\right]$ (*ξ* = 1, 2,…, *h*) then forms, where ***f*** and ξ respectively denote the *fitted data* and the *data number* in this matrix. The median value of the *h* data on the fitted centerline is then regarded as a sampled value of the groove center.

Overall, SCVR can self-adaptively recognize such variation coefficients as CV_00_, CV*_T_*, ${\mathrm{CV}}_{\min}^{\left(L\right)}$, and ${\mathrm{CV}}_{\min}^{\left(G\right)}$, thereby applying an adaptive filter to detect the groove center after adaptively adjusting the data window height. Compared to the commonly used value of ≥80 × 80 pixels in previous studies [16,26], SCVR can reduce the original ROI size to 60 × 60 pixels to improve the detection efficiency. Additionally, SCVR integrates global and local variation coefficient recognitions for accurate detection of the real groove centerline by the adaptive bandpass filtering. This novel approach is independent of the directionality of disturbed data along the original edge or line, forming an adaptive pattern recognition method. Therefore, the SCVR method has potential applications in edge detection beyond the field of welding, offering broader applicability.

#### 3.2.2. Dynamic Solution of SCVR Algorithm

**Figure 5:**
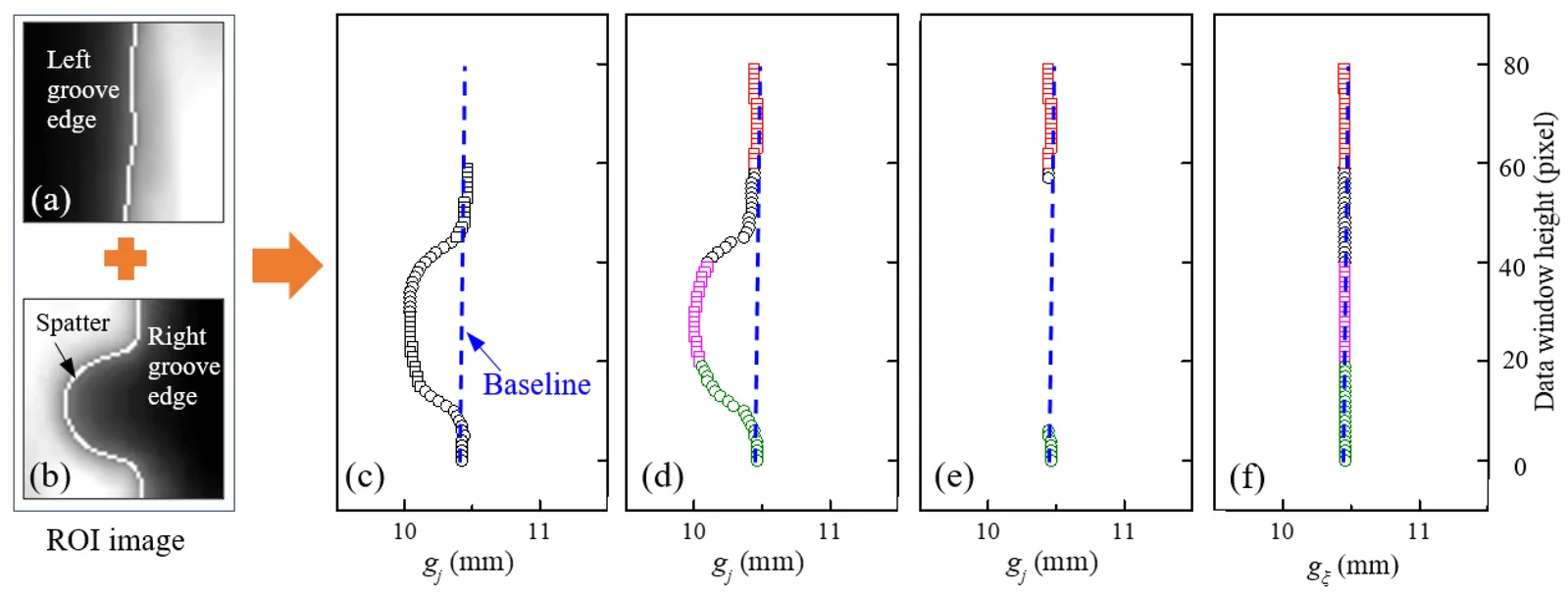
Dynamic solution procedure of SCVR algorithm. (**a**) ROI image of left groove edge. (**b**) ROI image of right groove edge with actual spatter. (**c**) Initial raw data distribution [*g_j_*] of groove centerline containing the spatter interference. (**d**) Raw data distribution [*g_j_*] of groove centerline containing the spatter interference. (**e**) Real data distribution [*g_j_*] in situ remained on groove centerline. (**f**) Reconstructed data distribution [*g_ξ_*] of groove centerline.

To facilitate understanding of the SCVR algorithm, Figure 5 illustrates the dynamic solution procedure of the SCVR method. In this figure, a blue dashed line denotes a manually chosen baseline of the real groove centerline for evaluating the reconstructing accuracy of the centerline. Additionally, two original ROI images, each with a size of 60 × 60 pixels, are shown in Figure 5a,b, intercepted respectively from Figure 2a,b. In Figure 5b, a large actual welding spatter with a ~1.2 mm diameter is clearly attached onto the right edge of the groove. After processing the images as described in Section 2.2, a raw data distribution matrix, $C_{j}^{r}$ = $\left[g_{j}\right]$, of the groove centerline is established within the data window of *h*_0_ (*h*_0_ = 60 pixels). This distribution exhibits a significant leftward bulge due to the spatter interference, as indicated in Figure 5c.

To assess the suitability of the initial height, *h*_0_, of the data window, the variation coefficient, CV_00_, is calculated for the initial raw data distribution in Figure 5c. Since CV_00_ is calculated as 0.01704, exceeding the set value, CV*_T_*, of 0.006, the data window height is increased self-adaptively by a set increment, *h*_c_ = 20 pixels. Accordingly, the data window height, *h*, becomes (*h*_0_ + *h*_c_), completing *Step* 1 of the SCVR algorithm. Note that the set values of CV*_T_* and *h*_c_ will be validated in Section 3.2.3 and Section 3.3.

In *Step* 2, the raw data distribution matrix $C_{j}^{r}$ = $\left[g_{j}\right]$ is optimally divided into 4 equal segments along the window height to avoid the local optima during the global search for ${\mathrm{CV}}_{\min}^{\left(L\right)}$, as color-coded in Figure 5d. In this case, the height, *h*/*m*, of each segment is 20 pixels. The variation coefficients, ${\mathrm{CV}}_{\varepsilon }$ (*ε* = 1, 2, 3, 4), are then calculated from the top to the bottom for each segment in Figure 5d, yielding values of 0.00092, 0.01046, 0.00268, and 0.01475, respectively. The minimum coefficient of variation, ${\mathrm{CV}}_{\min}^{\left(L\right)}$ = 0.00092, is recognized in the 1st segment. Consequently, the bandwidth, *D*_width_, of the in situ bandpass data filter is determined by the cutoff values [10.44231, 10.46154], based on the 1st data segment.

Then, this filter removes the 3rd data segment and the portions of the 2nd and 4th data segments, which are primarily disturbed by the spatter. The preserved data matrix $C_{\eta }^{p}$ = $\left[g_{j}\right]$ constitutes a real data distribution in Figure 5e. Finally, corresponding to *Step* 3 of SCVR, the fitted data distribution matrix $C_{\xi }^{f}$ = $\left[g_{\xi }\right]$ for the groove centerline is derived, as shown in Figure 5f. This fitted data distribution closely aligns with the baseline, demonstrating the effectiveness of reconstructing the groove centerline.

#### 3.2.3. Adaptability of SCVR Algorithm

In narrow-gap GMAW, welding spatter adhering to the narrow groove sidewalls is a primary disturbance for groove-centerline detection. The other disturbances, such as the remaining welding fumes and the arc light after image preprocessing and the uneven illumination of the moving molten pool, can also cause deviations in detection. The adaptability of the SCVR algorithm is validated below for different disturbances.

**Figure 6:**
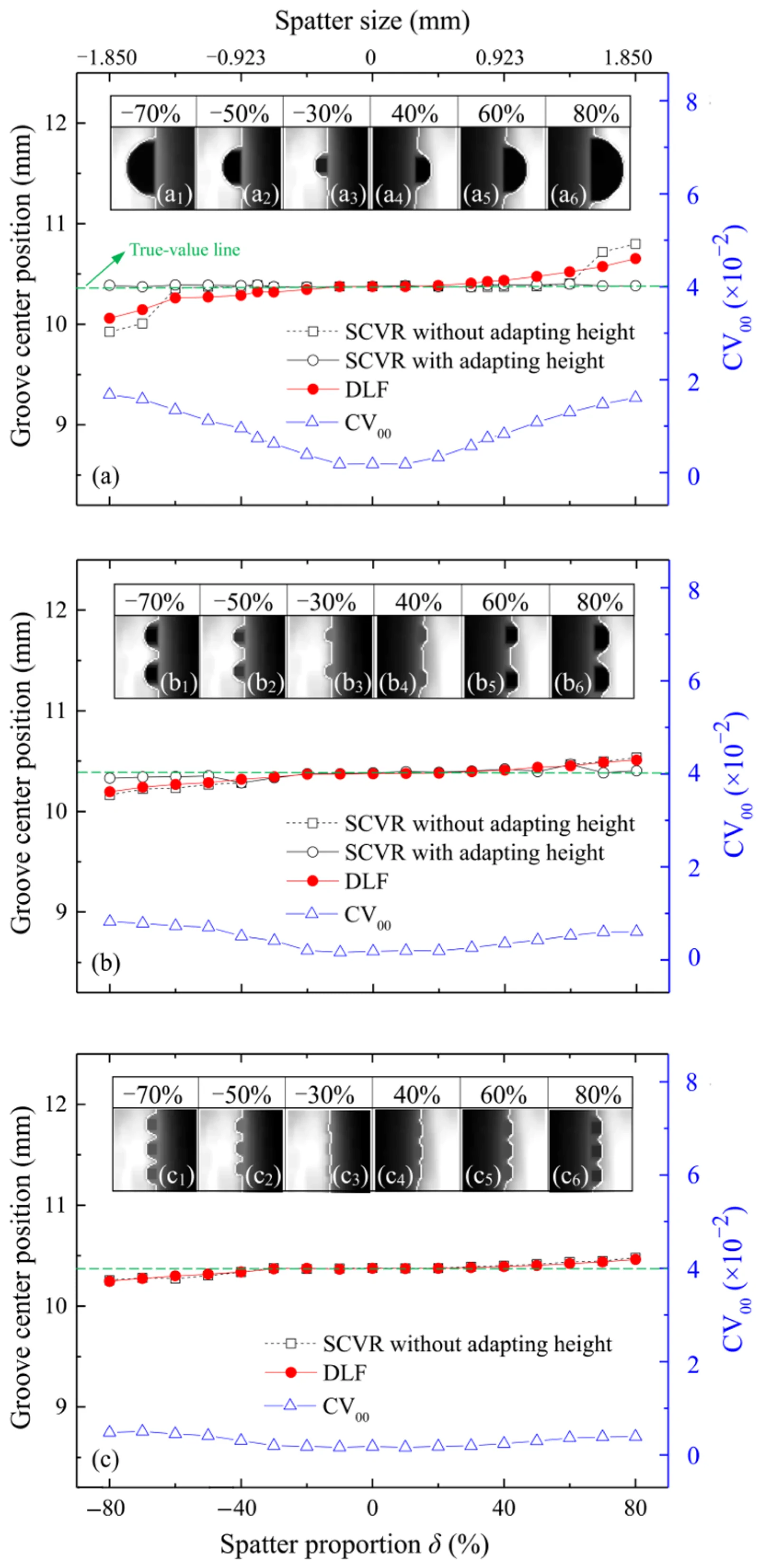
Effects of simulated-spatter number, *n*; size; and proportion on the variation coefficient, CV_00_, and the sampled value of the groove center determined by the SCVR and DLF methods. (**a**) *n* = 1. (**b**) *n* = 2. (**c**) *n* = 3.

**(a) Effects of varying-level spatters**. Figure 6 applies different methods to analyze the influence of spatter number, size, and proportion on the variation coefficient, CV_00_, and the sampled value of the groove center. These approaches include the SCVR methods with and without adapting height and the DLF (direct linear fitting) method.

In Figure 6a–c, the spatter numbers are 1, 2, and 3 with the same *δ*, and the spatter size corresponds to the proportion *δ*. Here, CV_00_ is calculated from the initial raw data distribution of the groove centerline by averaging the coordinates of the left and right groove edges, which are extracted from the ROI images of 60 × 60 pixels. Additionally, one groove edge is embedded with a set of simulated spatters at prescribed proportions, |*δ*| = 0%, 10%, …, 80%, while the opposite edge is kept free of spatter (*δ* = 0%). The proportion, *δ*, is defined as

(7) $$\delta \;=\;(n\cdot \;d_{s})/h_{0}$$

where *d_s_* denotes the diameter of the simulated spatter and *n* is the number of the simulated spatter; the negative and positive values of *δ* indicate that the spatter is on the right and left edges of the groove, respectively.

To create ROI images with simulated spatters of different sizes, the original ROI images of right and left groove edges were intercepted from the welding images in Figure 1a and Figure 2a. A group of simulated semicircular spatters were then pasted onto one groove edge of each welding image. Partial examples of the ROI images are given in Figure 6(a_1_–a_3_), Figure 6(b_1_–b_3_), and Figure 6(c_1_–c_3_) for the right edge and in Figure 6(a_4_–a_6_), Figure 6(b_4_–b_6_), and Figure 6(c_4_–c_6_) for the left edge, corresponding to the size proportions of −70%, −50%, and −30%, as well as 40%, 60%, and 80%, respectively.

As can be observed in Figure 6, CV_00_ increases with the absolute value, |*δ*|, of the spatter proportion and decreases with the spatter number, *n*, regardless of the spatter on the right or left edge of the groove. With intensifying this disturbance for SCVR without adapting height or DLF, the sampled values for the groove center gradually deviate from the true-value green dashed line. For clear presentation, two types of sampled values are shown with the black dash-square-dotted and red lines.

Typically, in Figure 6c, SCVR does not need to activate the height-adapting mechanism when sampling the values for the groove center, where the values closely match the ones by DLF. Compared to the true value sampled by DLF at |*δ*| = 0, the values sampled by SCVR without adapting height fluctuate merely within the range of −0.120 ~ +0.105 mm at the spatter number *n* = 3. This accuracy is very satisfactory even without the adaptive height adjustment. In this case, the maximum value of CV00 is 0.00502, which can be selected as CV_*T*_ in SCVR, setting CV_*T*_ to be 0.006. Accordingly, the set value of CV_*T*_ in Section 3.2.2 is justified.

For spatter numbers of *n* = 1 and 2, as shown in Figure 6a,b, the detection error of SCVR without adapting height reaches the ranges of −0.427~+0.466 mm and −0.213~+0.160 mm, respectively. To improve the detection accuracy, SCVR needs to activate its adaptive mechanism, increasing the data window height by *h_c_* = 20 pixels, resulting in *h* = (*h*_0_ + *h_c_*) = 80 pixels, as CV_00_ exceeds the set CV*_T_* value of 0.006. This adaptive adjustment occurs when *δ* is approximately smaller than −30% and −50% and exceeds 40% and 70% respectively on the right and left edges of the groove in Figure 6a,b. As a result, SCVR with adaptive adjustment, denoted by the black solid-circle-dotted line in Figure 6a,b, accurately detects the groove center with an absolute detection error of ~0.1 mm at *m* = 4.

The above results reveal that the diameter and proportion of the spatter affect the data variation and the detection accuracy. SCVR effectively counters this influence by triggering a timely adaptive adjustment of the data window. This simultaneously demonstrates a strong resistance of SCVR to the welding spatter, even at a proportion of 80%, corresponding to the spatter with an ~1.85 mm diameter. Therefore, SCVR shows an excellent adaptability to noisy environments with simulated spatters of varying number, size, and proportion.

**Figure 7:**
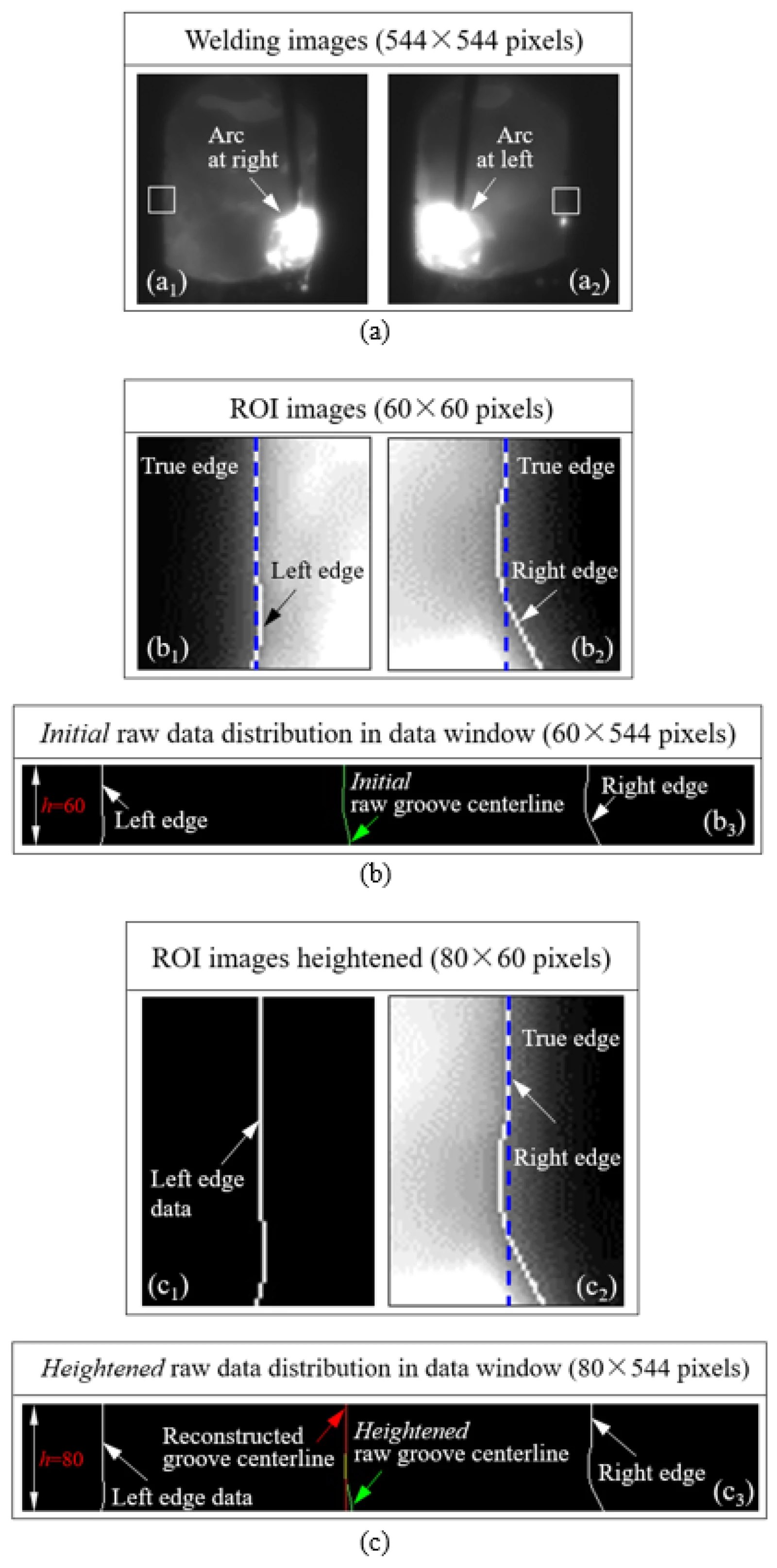
Effects of welding fumes and arc light. (**a**) Welding images: (**a_1_**,**a_2_**) welding images at the right and left positions of the arc. (**b**) Initial case: (**b_1_**,**b_2_**) ROI images of the groove edges and (**b_3_**) initial raw data distribution of the groove centerline. (**c**) Heightened case: (**c_1_**,**c_2_**) heightened ROI images and (**c_3_**) heightened raw data distribution of the groove centerline after heightening.

**Figure 8:**
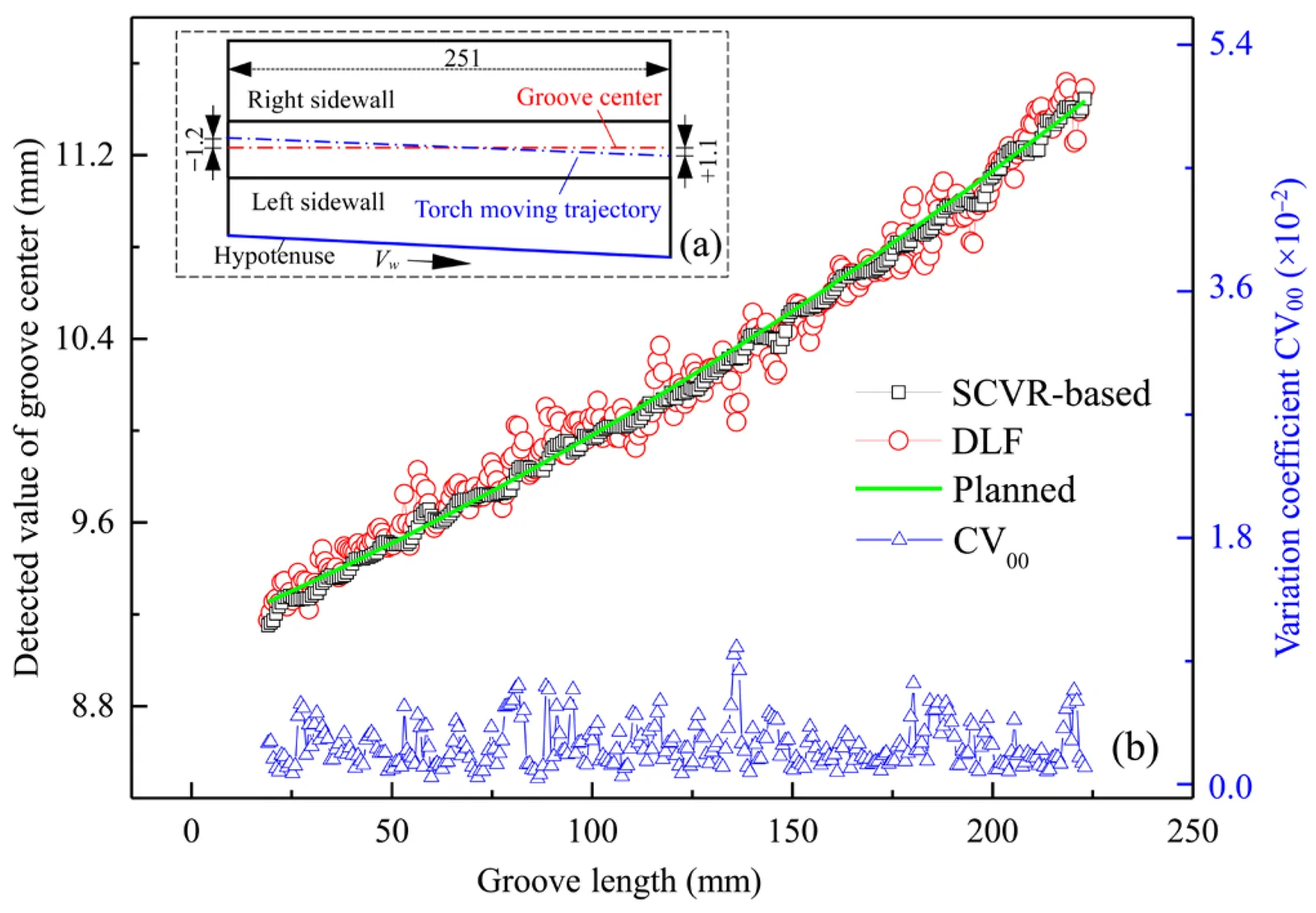
Groove-center detection results obtained by the DLF and SCVR-based methods. (**a**) Testpiece illustration. (**b**) Detection results.

**(b) Effect of welding fumes and arc light**. In addition to welding spatters, the arc light and welding fumes distort the groove edge extracted during image processing. Figure 7 shows such an instance as the torch deviates towards the right edge of the groove, which corresponds to the weld deviation of −1.06 mm in Figure 8. In this case, a depression-type disturbance with a depth of up to 0.36 mm occurs due to the light reflection from the arc.

Figure 7(a_1_) and (a_2_), (b_1_) and (b_2_), and (c_1_) and (c_2_) are welding images at the stays of the arc near the right and left sidewalls and the initial and heightened ROI images of the left and right groove edges, respectively. Figure 7(b_3_,c_3_) show the data distributions of the left and right edges and the initial/heightened raw groove centerline in the data window. In Figure 7(b_2_), the white right edge of the groove significantly curves, due to the nonuniform image grayscale distribution. This results from the welding fumes and the reflection of the arc light on the right sidewall of the groove.

This disturbance further propagates to the groove centerline shown in Figure 7(b_3_), which leads to a value of 0.00651 for the variation coefficient, CV_00_, in the initial raw data distribution of the centerline. Since this value is greater than the set value 0.00600 of CV*_T_*, the height of the data window increases from 60 pixels in Figure 7(b_3_) to 80 pixels in Figure 7(c_3_), forming the heightened raw data distribution of the centerline.

As the adaptive SCVR method is applied to the raw groove centerline indicated by the green line in Figure 7(c_3_), a real groove centerline is accurately reconstructed as shown by the red line in Figure 7(c_3_). SCVR effectively suppresses the disturbances and gains the true position of the groove center.

The simulated spatters provide a controllable and repeatable means to systematically quantify the influence of the spatter number, size, and proportion, whereas the real welding spatter (Figure 3f and Figure 5b) and the welding fumes and arc light (Figure 7) verify the effectiveness of SCVR against the actual, time-dependent disturbances in practical welding. These simulated and real disturbances thus provide a complementary validation of the adaptability of SCVR.

### 3.3. Weld Deviation Dectection Results Obtained by SCVR

#### 3.3.1. Detection of Groove Center

To detect weld deviation from welding images, accurate detection of the groove center is crucial, as the torch position in the images remains almost unchanged due to the camera being attached to the torch. Figure 8 compares the groove-center detection results obtained by different algorithms.

Figure 8a provides a top view of the testpiece, which was directly machined from a mild steel plate with a thickness of 50 mm and a length of 251 mm. A square groove with a depth of 20 mm and a width of 13.8 mm is located in the center of the ending width of the testpiece, with its centerline parallel to the length direction of the testpiece. Additionally, the testpiece, with an initial width of 51.2 mm and a final width of 53.5 mm, has a hypotenuse ratio of 2.3:251. During welding, the testpiece moves relative to the torch along the hypotenuse at a welding speed of *V_w_*, yielding a preset weld deviation of −1.2~+1.1 mm. The other experimental parameters are the same as those in Table 1 and Table 2, except that the arc swing angle is 72°. The setting of the smaller angle is attributed to the presence of a torch deviation from the groove center at the beginning of the groove.

Figure 8b shows the groove-center detection results. The green line is derived from a statistical polynomial fitting of all the initial raw data distributions of the groove centerline along the groove length and thus represents the dynamically planned centerline of the groove, where each distribution contains *h*_0_ data points. This line, incorporating 301 × *h*_0_ (*h*_0_ = 60 pixels) raw data points, captures the dynamic changes in the real groove center during actual welding and serves as the reference line for evaluating the detection accuracy of the groove center.

In Figure 8b, the red circles and black squares denote the 301 detection values for the groove center obtained by the DLF and SCVR-based methods, respectively. To yield these detection values for each method, 302 frames of welding images were captured in real time across the effective groove length of 205.36 mm out of a total length of 251 mm. The blue line with triangles indicates noticeable fluctuations in CV_00_ due to welding noise, where 14 instances exceed the CV*_T_* threshold of 0.006. Among these, this fluctuation is particularly noticeable around lengths of ~81 mm, ~117 mm, ~137 mm, and ~220 mm, caused by disturbances from welding spatter, arc light, and welding fumes. In these cases, SCVR adaptively increases the height of the data window by 20 pixels.

As a result, by filtering out the disturbed values from the raw data distribution, the SCVR-based method accurately detected the groove-center values with a small error range of −0.121~+0.065 mm relative to the green reference line. In contrast, the DLF method produced detection values that varied within a much larger error range of −0.324~+0.261 mm, as it lacks the capability to remove welding interference. The accurate detections by SCVR suggest that the above selections of the data segment number *m* and the adaptive height increment *h_c_* are suitable for actual welding.

#### 3.3.2. Detection of Weld Deviation

**Figure 9:**
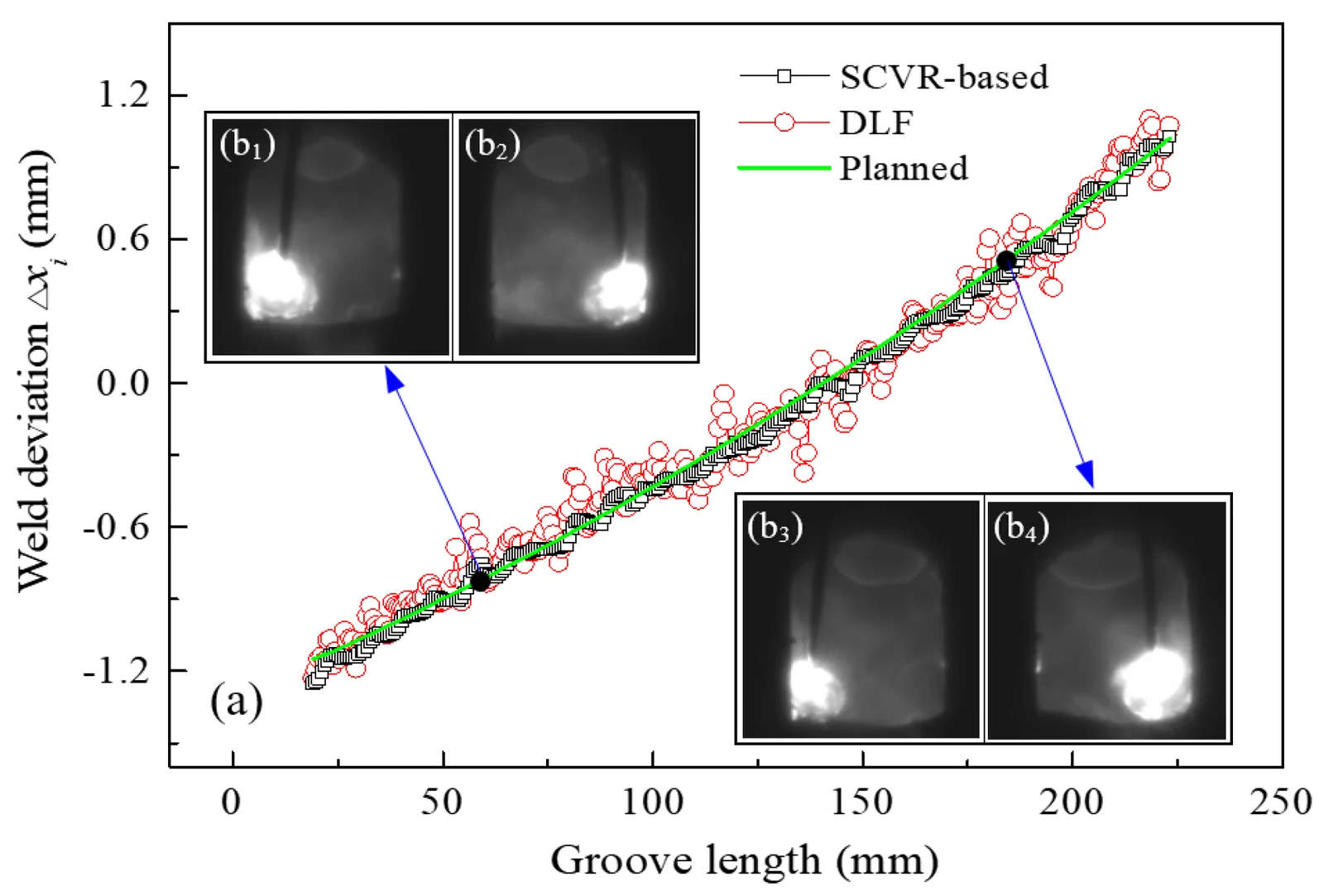
Detection results for weld deviation. (**a**) Comparison of detected weld deviation values with the planned ones. (**b_1_**,**b_2_**) Welding images with the arc stayed near the left and right sidewalls at the groove length of ~65 mm. (**b_3_**,**b_4_**) Welding images with the arc stayed near the left and right sidewalls at the groove length of ~185 mm.

To obtain a detection value, Δ*x_i_*, for the weld deviation, the groove-center position detected by the above DLF and SCVR-based methods is compared with the torch position, which is detected using the PCF algorithm from [16]. Figure 9 shows the detection results, where the green line indicates the planned weld deviation. The planned deviations are calculated by finding the difference between the planned values for the groove center in Figure 8b and the PCF-detected values for the torch position.

Compared to the DLF method, the SCVR-based method more accurately detects weld deviation, as indicated by the red and black points in Figure 9a. The deviation detection accuracies of the two methods are nearly the same as those for groove-center detection because the PCF-detected torch position remains almost unchanged. Considering the dispersion of the error values of the detected weld deviation, the SCVR-based method results in a smaller standard deviation of 0.0324 compared to 0.0884 for the DLF method. Furthermore, in comparison to the most relevant ODF-based method in [16], the SCVR-based method reduces the detection time for weld deviation by ~17%, in addition to simplifying the detection algorithm. These results demonstrate that the SCVR-based method can accurately and efficiently detect weld deviation during actual narrow-gap welding, thereby facilitating real-time tracking control.

In addition, to visually display the arc state within the narrow-gap groove at different weld deviations, two pairs of adjacent welding images were acquired at groove lengths of ~64 mm and ~185 mm while the arc swung to shortly stay near the left and right sidewalls of the groove, as shown in Figure 9(b_1_,b_2_), as well as Figure 9(b_3_,b_4_). The arc distinctly deviated toward the right and left sidewalls of the groove, as indicated in Figure 9(b_2_,b_3_), with the planned deviation values of −0.769 mm and +0.523 mm, respectively. In other words, the presence of the weld deviation led to the unequal distances between the arc and the two groove sidewalls, which lowers welding quality. Hence, weld tracking is essential to ensure consistent weld formation.

## 4. Weld Tracking Experiments

### 4.1. Control Strategy

**Figure 10:**
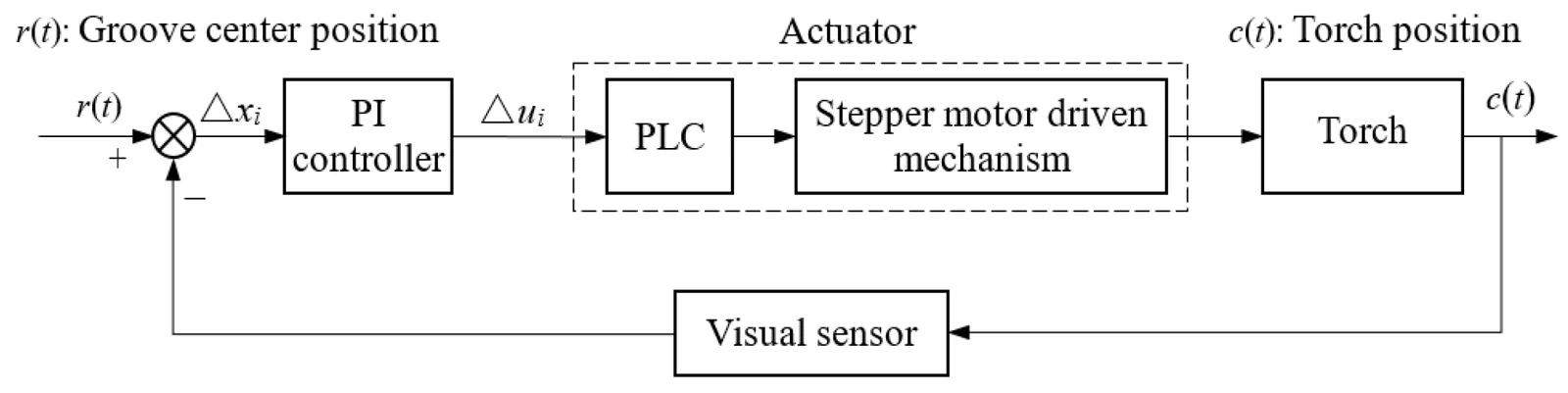
Block diagram of infrared-vision-based real-time tracking control system for narrow-gap welding.

Figure 10 is a block diagram of the infrared-vision-based real-time tracking control system for swing-arc narrow-gap welding, where the groove-center position, *r*(*t*), and the torch position, *c*(*t*), are respectively the given and output values of the system. Its core components include a PI (proportional–integral) controller, an actuator, a visual sensor, and a comparator.

During control, the visual sensor detects a torch position using the PCF algorithm and adaptively extracts a value for the groove center via the developed SCVR algorithm. The comparator then calculates the difference between the groove-center position, *r*(*t*), and the torch position, *c*(*t*), to yield a weld deviation, Δ*x_i_*. Subsequently, the PI controller utilizes Δ*x_i_* to produce a current adjustment value, Δ*u_i_*, which is transmitted to a PLC in the actuator via the RS232 interface. This PLC finally drives the stepper motor-based mechanism in the actuator to correct the torch position, realizing the weld tracking by compensating the deviation, Δ*x_i_*. The above related image processing and control algorithm is run on a computer. Such a distributed control system, incorporating a PLC-based actuator with a computer-based PI controller, offers a rapid task response due to the parallel control.

The tracking system is a digital closed-loop servo system with a feedback coefficient of 1.0. Additionally, its actuator contains a stepper motor mechanism with a position-maintaining function, making the actuator generally regarded as an inertial link with delay. Consequently, an incremental PI controller is adopted and expressed as

(8) $$\Delta u_{i}=P[({\Delta x}_{i}\;-\;{{\Delta x}_{i-1})+I\cdot \Delta x}_{i}]$$

where *P* and *I* represent the proportional and integral coefficients of the PI controller and ${\Delta x}_{i}$ and ${\Delta x}_{i-1}$ denote the current and previous values for weld deviation. Based on numerical simulations and preliminary experiments, the values of *P* and *I* are tuned respectively as 0.5 and 0.3, obtaining the PI controller with optimal stability. The above control strategy will be further validated by actual tracking experiments.

### 4.2. Tracking Results

#### 4.2.1. Experimental Procedure

**Figure 11:**
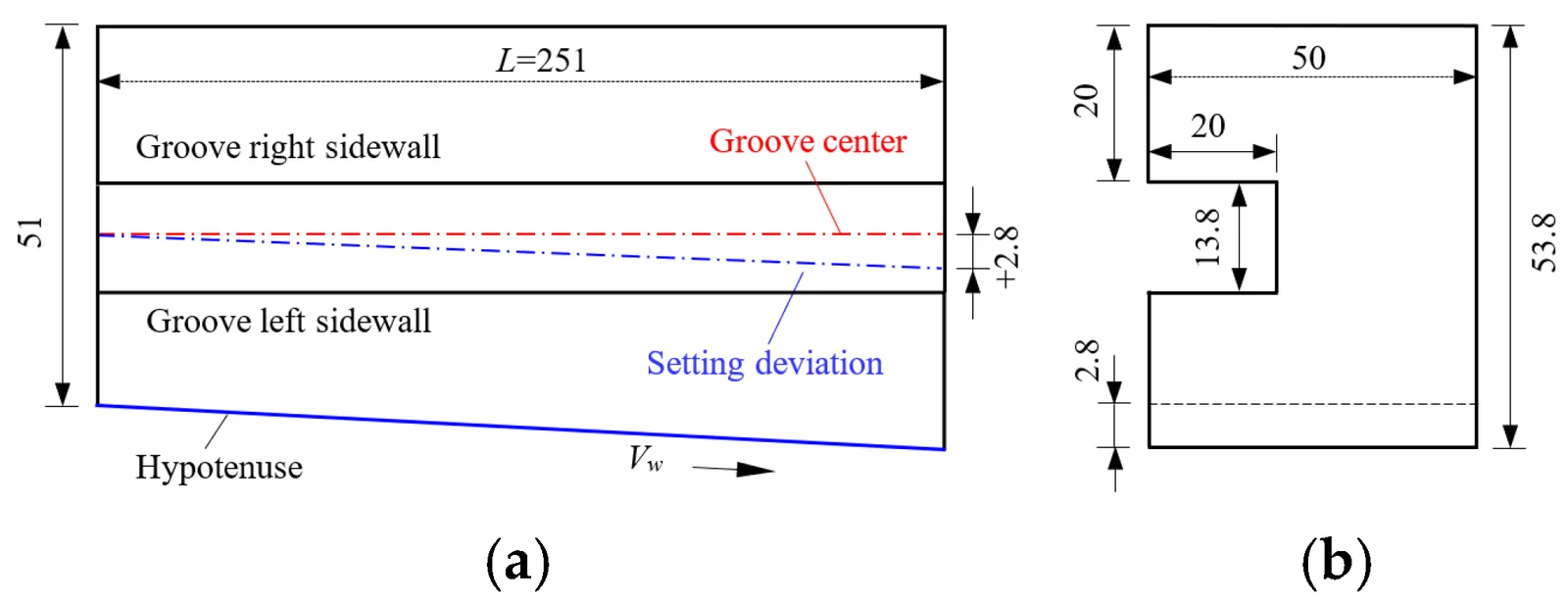
Illustration of testpiece. (**a**) Top view. (**b**) Right view.

To verify the effectiveness and real-time control accuracy of the weld tracking method, a number of infrared visual sensing weld tracking control experiments were carried out. The pulsed welding experimental conditions are the same as those in Table 1 and Table 2, except for the testpiece. Figure 11a,b show the top and right views of the testpiece, respectively. This testpiece had an initial width of 51 mm and a final width of 53.8 mm, resulting in a preset weld deviation of 2.8 mm due to a hypotenuse ratio of 2.8:251. All the other conditions of the testpiece are the same as those indicated in Figure 8a.

#### 4.2.2. Weld Deviation Results Analysis

**Figure 12:**
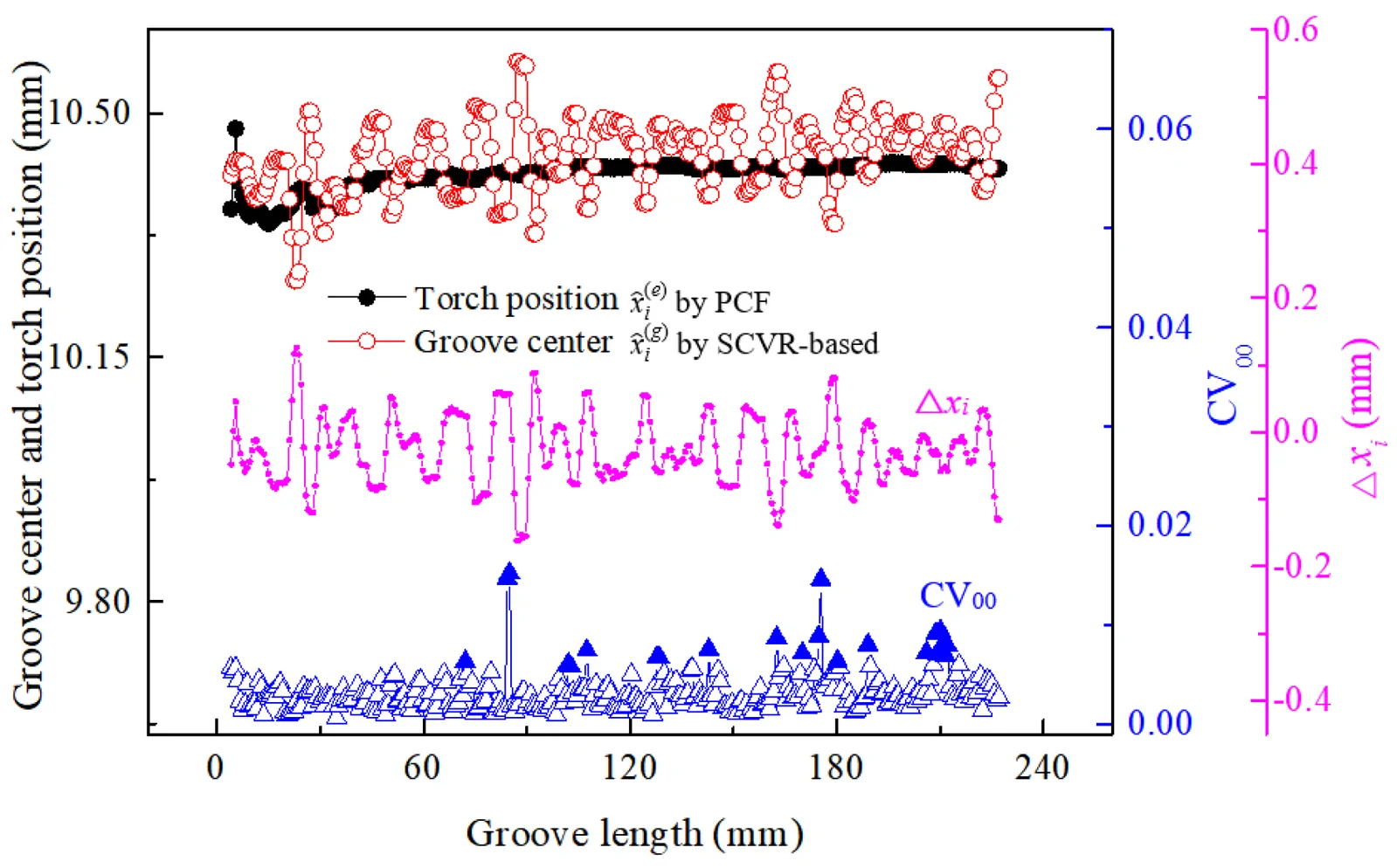
Detection results for groove center; torch position; CV_00_; and weld deviation, Δ*x_i_*, during real-time weld tracking.

Figure 12 shows the detected examples of groove center, torch position, CV_00_, and weld deviation during real-time weld tracking. As the arc entered into the groove, welding images were continuously captured to detect and compensate for the weld deviation until the arc was extinguished. The sampling rate of the image was set as half of the arc swing frequency, i.e., 1.25 Hz.

Along the effective groove lengths of 4.08~227.12 mm, 329 values for groove center, ${\hat{x}}_{i}^{\left(g\right)}$, and torch position, ${\hat{x}}_{i}^{\left(e\right)}$, were synchronously detected from 330 adaptively intercepted frames of ROI images, as shown respectively by the red and black lines in Figure 12. Relative to the camera, the PCF-based [16] detection values for the torch position always changed within a very small range, with a maximum absolute error of only 0.087 mm. In contrast, the SCVR-based detection values for the groove center varied between 10.261 and 10.574 mm, with a maximum absolute error of 0.313 mm.

The resulting weld deviation during closed-loop control is shown by the purple line in Figure 12. The PI controller achieved high tracking accuracy within the range of −0.161~+0.126 mm, despite a small average static tracking error of 0.028 mm (see the red and black lines in Figure 12).

In Figure 12, the blue triangle denotes the coefficient of variation (CV_00_) for the initial raw groove-center data distribution, where CV_00_ ranges from 0.000679 to 0.01525. Among these values, 21 values for CV_00_ are greater than the set CV*_T_*, as indicated by the blue solid triangles. During the weld tracking, SCVR consistently operated in an adaptive mode. Notably, in the 21 cases where CV_00_ exceeded CV*_T_*, SCVR adaptively increased the height of the data window from the original height, *h*_0_ (see Figure 4), of 60 pixels to the height, *h*, (see Figure 4) of 80 pixels so as to adapt the high level of the welding interference. This adaptive mechanism ensured accurate detection of the weld deviation by the SCVR-based algorithm.

#### 4.2.3. Tracking Adjustment Results Analysis

**Figure 13:**
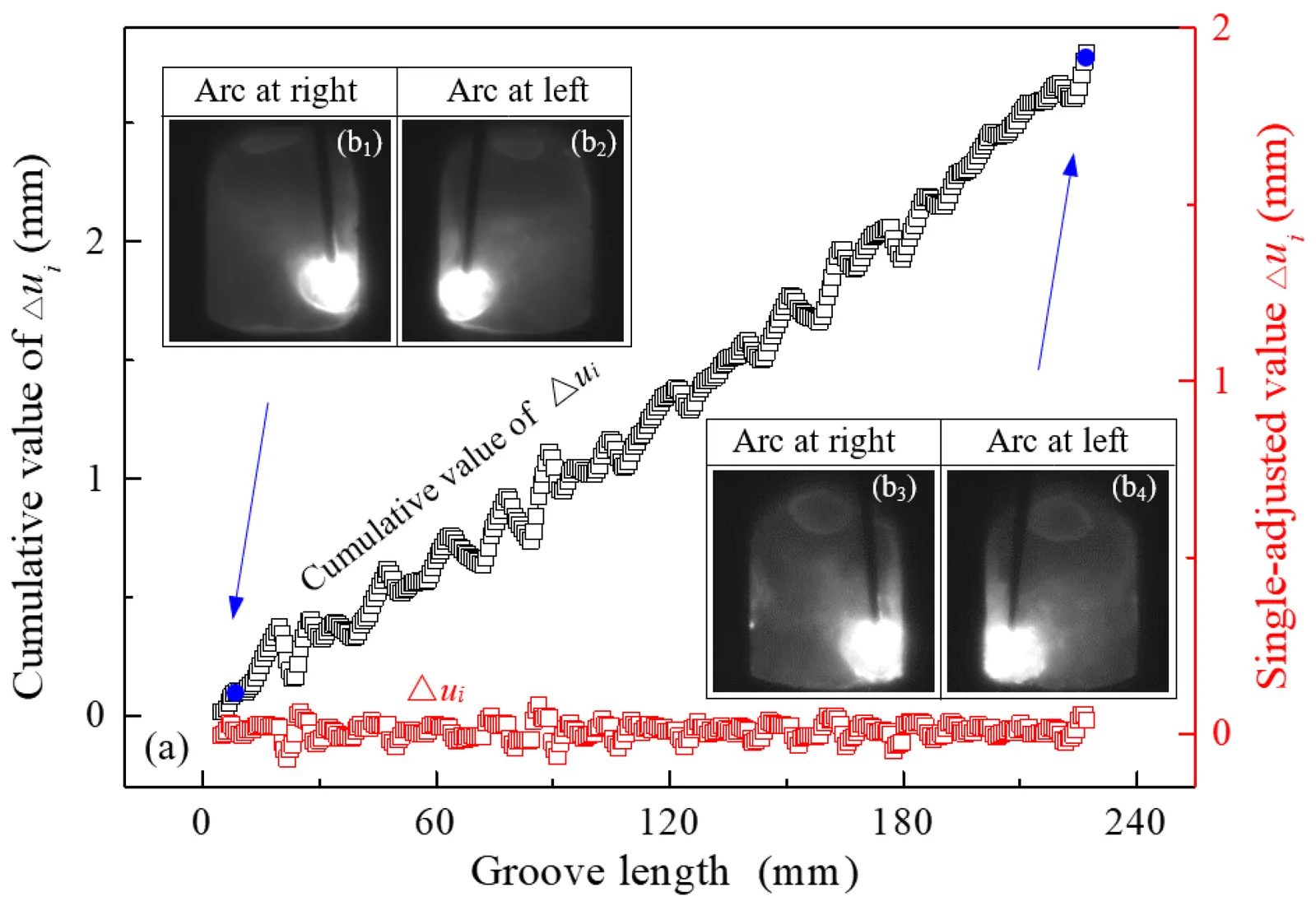
Results for single-adjusted value Δ*u_i_* and its cumulative value during real-time weld tracking. (**a**) Values for Δ*u_i_*. (**b_1_**–**b_4_**) Examples of welding images.

Figure 13 shows the real-time adjusted values for torch position, where the single-adjusted value, Δ*u_i_*, and the cumulative values for Δ*u_i_* are indicated by red and black squares. Accordingly, 329 compensations were completed during the weld tracking, with each adjustment taking less than 50 ms. This time primarily covers the weld deviation detection time of ~25 ms and the image transmission and deviation compensation time of <25 ms, using a computer with a 2.93 GHz processor.

Critically, this short adjustment time implies that the deviation adjustment process is accomplished while the arc stays near the sidewall, since the arc at-sidewall staying time is actually 100 ms when the arc swing frequency is 2.5 Hz. In other words, each deviation is completely compensated within the semi-circle 200 ms of the arc swing. This rapid response, together with the tracking accuracy indicated in Section 4.2.2, demonstrates the good real-time capability of the SCVR-based tracking system.

Additionally, the PI controller merely made a small adjustment, Δ*u_i_*, to the torch position relative to the groove at each regulation, with the maximum single adjustment of 0.083 mm. As every value of Δ*u_i_* is summed over the groove length, the torch position is cumulatively adjusted from 0.023 mm to 2.797 mm, with minor continuous fluctuations due to using the PI controller without a dead zone. The net total adjustment is accordingly 2.774 mm, which closely approximates the preset deviation of 2.534 mm over the effective groove length of 223.04 mm. The slight discrepancy is primarily attributed to welding thermal distortion. The above results reflect the effective tracking capability of the system, as well as its strong real-time performance and stability.

To demonstrate the actual state of the arc during the weld tracking, Figure 13(b_1_–b_4_) present two pairs of adjacent welding images at groove lengths of ~10.20 mm and ~227.12 mm, where the arc swung to shortly stay near the right and left sidewalls of the groove, respectively. It can be observed that the distance between the arc and each groove sidewall remained almost consistent, which is crucial for forming uniform sidewall penetration and ensuring sound weld formation.

#### 4.2.4. Tracking Effect Results Analysis

**Figure 14:**
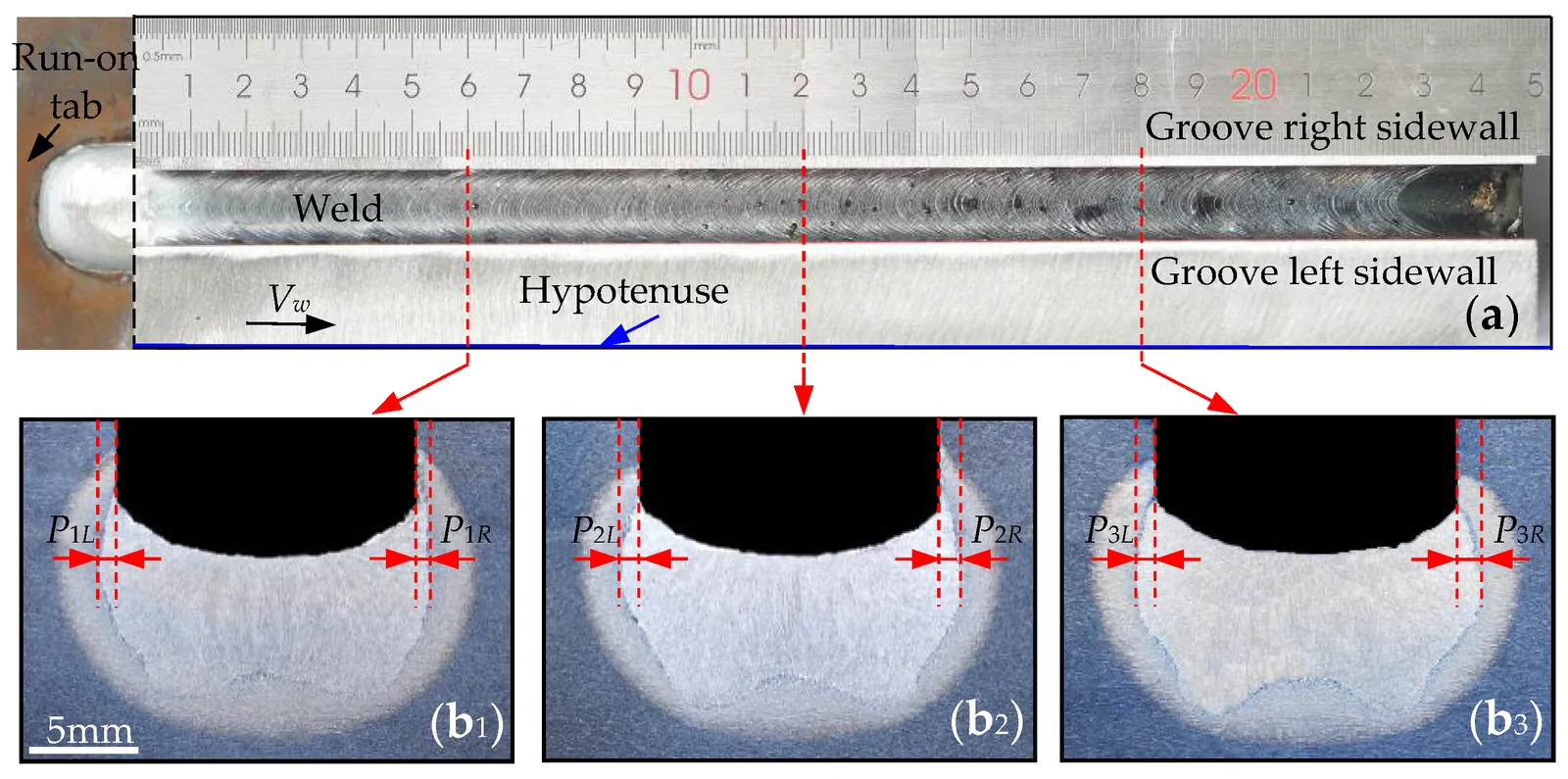
Photographs of welding bead formation. (**a**) Weld appearance. (**b_1_**–**b_3_**) Bead cross-sections at the distances of 60 mm, 120 mm, and 180 mm from the start of the groove.

Finally, Figure 14 shows the photographs of the final weld appearance and bead cross-sections. Here, *P*_1*L*_ and *P*_1*R*_, *P*_2*L*_ and *P*_2*R*_, and *P*_3*L*_ and *P*_3*R*_ denote the penetration depths in the left and right sidewalls of the groove at the distances of 60 mm, 120 mm, and 180 mm from the start of the groove, respectively. Obviously, the roughly symmetrical penetrations of the two sidewalls formed on each cross-section of the bead, while good surface formation was exhibited. The above experimental results demonstrate that the weld tracking contributed to the stable welding and verify the effectiveness of the developed detection algorithms and control system during the weld tracking.

### 4.3. Advantages of the SCVR-Based Approach

Compared to other similar approaches, the proposed SCVR-based weld deviation detection and tracking control system demonstrates higher detection accuracy, efficiency, and adaptability, as summarized in Table 3.

In relation to comparable weld deviation detection algorithms [16,22,26], the SCVR algorithm presents four main advantages: (1) it is independent of the data distribution direction by recognizing the variation coefficient of the distribution; (2) it establishes a data window of adaptive height adjustment, removing the influence of the welding disturbance level on the detection accuracy; (3) it applies the low original height of 60 × 60 pixels of the ROI window owing to a height-adapting mechanism, obviously reducing the detection time consumption by 17% compared to the most relevant algorithm [16]; (4) it designs an in situ bandpass data filter with adaptive bandwidth, realizing a globally optimal selection of the real data after filtering out the disturbed data. Furthermore, the SCVR-based tracking control system enables weld tracking with an accuracy of −0.161~+0.126 mm. This accuracy is within the appropriate range of around ±0.2 mm for the industrial welding in the deep and narrow groove and outperforms the precision of ±0.47 mm of the most relevant method [25].

To sum up, the developed method was tested on ~700 frames of the welding images during the adaptability test for varying-level interference, the offline weld deviation detection test, and the real-time weld tracking experiments. The test results demonstrate the effectiveness and adaptability of our algorithm against different types of welding interference.

## 5. Conclusions

(1) A Self-adaptive Coefficient-of-Variation Recognition (SCVR) algorithm is proposed to adaptively detect the groove center from infrared images in swing-arc narrow-gap GMAW. SCVR incorporates an in situ bandpass data filter and adaptively determines the filter bandwidth by locally searching for the data segment with the minimal variation coefficient in the raw groove-centerline data distribution. This filter then globally minimizes the variation coefficient of the remaining in situ data, probably across different data segments of the distribution, by excluding disturbed data. This enables SCVR to accurately recognize the real groove center from the filtered data, enhancing the algorithm’s adaptability while greatly simplifying the detection process.

(2) A data window of self-adaptive height is built to reduce the proportion of the disturbed data within the raw data distribution, enabling the formation of an appropriate bandpass for the data filter in SCVR. The window height accordingly increases self-adaptively by a given value, *h_c_*, when the variation coefficient exceeds a set threshold, CV*_T_*. Both simulated-spatter and actual welding experiments confirm that CV*_T_* = 0.006 and *h*_c_ = 20 pixels are appropriate values. SCVR with adaptive height adjustment can filter out spatter up to 1.85 mm in diameter, offering strong resistance to large welding spatters. Additionally, weld deviation is detected more quickly, as SCVR enables the use of a smaller 60 × 60 pixel ROI, increasing speed by ~17% compared to the previous algorithms. This thus increases the detection efficiency while enhancing the algorithm’s robustness.

(3) A real-time weld tracking control system for swing-arc narrow-gap GMAW, incorporating a PLC-based actuator with a PI controller, has been developed based on infrared vision. It utilizes the adaptive SCVR approach with strong anti-interference ability to achieve high weld-deviation detection accuracy within a range of −0.121~+0.065 mm. Weld tracking is stably realized with a precision of −0.161~+0.126 mm and a regulation time of <50 ms. As a result, high-quality welds with nearly symmetrical penetration into both sidewalls of the groove are produced. These results demonstrate the effectiveness of the developed algorithm and system.

Overall, the present work addressed weld deviation detection and weld tracking with high accuracy in single-layer narrow-gap welding. This high accuracy is attributed to three main factors: the visual sensing with high precision, the adaptive detection algorithm with high accuracy, and the control system with good real-time capability. Actually, in multi-layer welding, partial spatters of different sizes and numbers remain on the groove sidewalls after former-layer welding. In future work, we are planning to investigate whether former-layer spatters influence the SCVR-based detection accuracy for subsequent-layer weld deviation. Moreover, since the passive surface imaging used here only observes the surface of the groove and the molten pool, we also plan to combine it with in situ X-ray imaging and multiphysics simulation [35,36] to reveal the internal molten-pool flow and the liquid–liquid interface evolution in narrow-gap welding.

## Figures and Tables

**Table 1:** Camera shooting parameters.

Name	Value
Wavelength of narrowband filter (nm)	970 ± 20
Transmittance of neutral density filter	30%
Aperture	*f*/16
Exposure time (ms)	0.2
Camera-to-wire distance (mm)	256
Angle of depression (*α*, °)	~30
Image size (pixel)	544 × 544

**Table 2:** Welding parameters.

Name	Value
Average arc current (A)	~320
Average arc voltage (V)	29~30
Arc current pulse frequency (Hz)	~250
Welding speed (*V_w_*, mm s^−1^)	3.4
Electrode wire diameter (mm)	1.2
Torch standoff height (mm)	20
Shielding gas/flowrate (L min^−1^)	Ar + 20%CO_2_/25
Groove gap (mm)	13.8
Swing frequency (Hz)	2.5
Swing angle (°)	82
Arc at-sidewall staying time (s)	0.1
Conductive-rod bending angle (°)	8

**Table 3:** Comparison of SCVR-based method with other relevant methods.

Algorithm	Purpose	Detection Accuracy (mm)	Detection Time (ms)	ROI Original Size (Pixel)/ Height Adapting (Yes/No)		Disturbance Level Limit
ODF [16]	Deviation detection	−0.107~+0.079	~30	80 × 80	No	Yes
Ref. [22]	Deviation detection	±0.3	~200	Height > 60	No	Yes
LPR [26]	Deviation detection	±0.086	~30	100 × 100	No	Yes
LMWR [25]	Weld tracking	±0.47	>25	Height > 60	No	Yes
SCVR (ours)	Deviation detection /weld tracking	−0.121~+0.065 /−0.161~0.126	~25	60 × 60	Yes	No

## Data Availability

Data are contained within the article.
